# Health Impact Modelling of Active Travel Visions for England and Wales Using an Integrated Transport and Health Impact Modelling Tool (ITHIM)

**DOI:** 10.1371/journal.pone.0051462

**Published:** 2013-01-09

**Authors:** James Woodcock, Moshe Givoni, Andrei Scott Morgan

**Affiliations:** 1 UKCRC Centre for Diet and Activity Research (CEDAR), Institute of Public Health, University of Cambridge, Cambridge, United Kingdom; 2 Department of Geography and the Human Environment, Tel-Aviv University, Tel Aviv, Israel; 3 Institute for Women's Health, University College London, London, United Kingdom; Fundación para la Prevención y el Control de las Enfermedades Crónicas No Transmisibles en América Latina (FunPRECAL), Argentina

## Abstract

**Background:**

Achieving health benefits while reducing greenhouse gas emissions from transport offers a potential policy win-win; the magnitude of potential benefits, however, is likely to vary. This study uses an Integrated Transport and Health Impact Modelling tool (ITHIM) to evaluate the health and environmental impacts of high walking and cycling transport scenarios for English and Welsh urban areas outside London.

**Methods:**

Three scenarios with increased walking and cycling and lower car use were generated based upon the Visions 2030 Walking and Cycling project. Changes to carbon dioxide emissions were estimated by environmental modelling. Health impact assessment modelling was used to estimate changes in Disability Adjusted Life Years (DALYs) resulting from changes in exposure to air pollution, road traffic injury risk, and physical activity. We compare the findings of the model with results generated using the World Health Organization's Health Economic Assessment of Transport (HEAT) tools.

**Results:**

This study found considerable reductions in disease burden under all three scenarios, with the largest health benefits attributed to reductions in ischemic heart disease. The pathways that produced the largest benefits were, in order, physical activity, road traffic injuries, and air pollution. The choice of dose response relationship for physical activity had a large impact on the size of the benefits. Modelling the impact on all-cause mortality rather than through individual diseases suggested larger benefits. Using the best available evidence we found fewer road traffic injuries for all scenarios compared with baseline but alternative assumptions suggested potential increases.

**Conclusions:**

Methods to estimate the health impacts from transport related physical activity and injury risk are in their infancy; this study has demonstrated an integration of transport and health impact modelling approaches. The findings add to the case for a move from car transport to walking and cycling, and have implications for empirical and modelling research.

## Introduction

Motorised transport is the fastest rising source of energy related greenhouse gas emissions and remains highly oil dependent [1]. Studies have suggested the potential for increased walking and cycling and reductions in car use to benefit population health and the environment [2], [3]. The largest potential health benefits have been identified as being from increases in physical activity, with additional benefits from reductions in air pollution but a potential increase in road traffic injuries.

The Visions 2030 Walking and Cycling project, (Visions 2030) considered alternative scenarios for the UK for the year 2030 in which walking and cycling play a central role in urban transportation. In this study we quantified scenarios for urban areas (population greater than 10,000 [4]) in England and Wales outside London based on Visions 2030 [5] and modelled their impact on population health and greenhouse gas emissions. London was excluded because travel patterns are different from other urban areas in the UK (higher public transport and lower car use) and because London was covered in an earlier study [2].

## Methods

Key assumptions used in this modelling study are shown in Table 1.

**Table 1:** Key assumptions in the health impact modelling.

Assumption	Sensitivity analyses
• Non-linear relationship between physical activity and health outcomes	• Alternative relationships tested
• Population physical activity treated as age group and sex specific log normal distributions with minimum threshold of 2.5 MET hours per week^1^	• Tested by comparison with HEAT models based on mean time spent walking and mean time spent cycling
• Road traffic injuries a non-linear product of distance travelled by each mode, average motor traffic speed, and baseline injuries for each pairwise combination of modes	• Linear model tested; exclusion of speed model tested
• Air pollution only modelled PM 2.5; assumed that reduction in emissions from road transport led to equal proportional reduction in primary PM concentrations attributed to road transport	• Assumed proportional reduction in national emissions from all sources led to proportional reduction in total concentrations (including primary and secondary sources)

PM 2.5 = particulate matter<2.5 nanometres diameter.

1 Median non-travel physical activity was added to travel physical activity for breast cancer, colon cancer, dementia, and depression but not for ischemic heart disease, cerebrovascular disease, or diabetes because walking specific relative risks were used for these three disease groups.

### Health Impact Modelling tool

This study used an Integrated Transport and Health Impact Model (ITHIM) to model the changes to population exposures of physical activity, air pollution and road traffic injury risk. ITHIM was developed out of the work published in Woodcock et al 2009 [2] and a similar model was used in Maizlish et al 2013 [6]. The spreadsheet is available from Dr James Woodcock on request.

ITHIM models physical activity exposures by comparing distributions of weekly physical activity under different scenarios. A comparative risk assessment method is used to estimate how changes in population physical activity and air pollution exposures result in changes in health outcomes. Road traffic injuries are modelled using a risk, distance and speed based model.

### Description of the Visions and scenarios

We developed three quantified scenarios based on the “Visions of the Role of Walking and Cycling in 2030” research [5] (see also http://www.visions2030.org.uk/) and compared these against a baseline scenario. For the baseline scenario data from a variety of sources, including the UK National Travel Survey, Transport Statistics for Great Britain, and Stats19, were used (see Table 2). In Vision 0 (baseline) individuals' mean walking time was 12.5 minutes per day and individuals' mean cycling time 0.9 minutes per day.

**Table 2:** Key data sources for baseline scenario.

Dataset	Years	Geographic coverage	Used for	Description of data
UK National Travel Surveys	2002–2008	People living in English and Welsh urban areas outside London	Person based travel (time, distance and speeds) Baseline ratios of walking time and cycling time to reference group	Self-reported weekly travel diary
Transport Statistics Great Britain	2002–2008	English and Welsh urban areas excluding London	Vehicle travel distances	Official statistics based on traffic counts and modelled flows
Stats19	2002–2008	English and Welsh urban areas excluding London	Serious injuries and fatalities by victim mode and striking vehicle	Police reported injuries and fatalities
Health Survey for England	2008	English and Welsh urban areas excluding London	Non-travel physical activity	Self-reported physical activity

**Figure 1:**
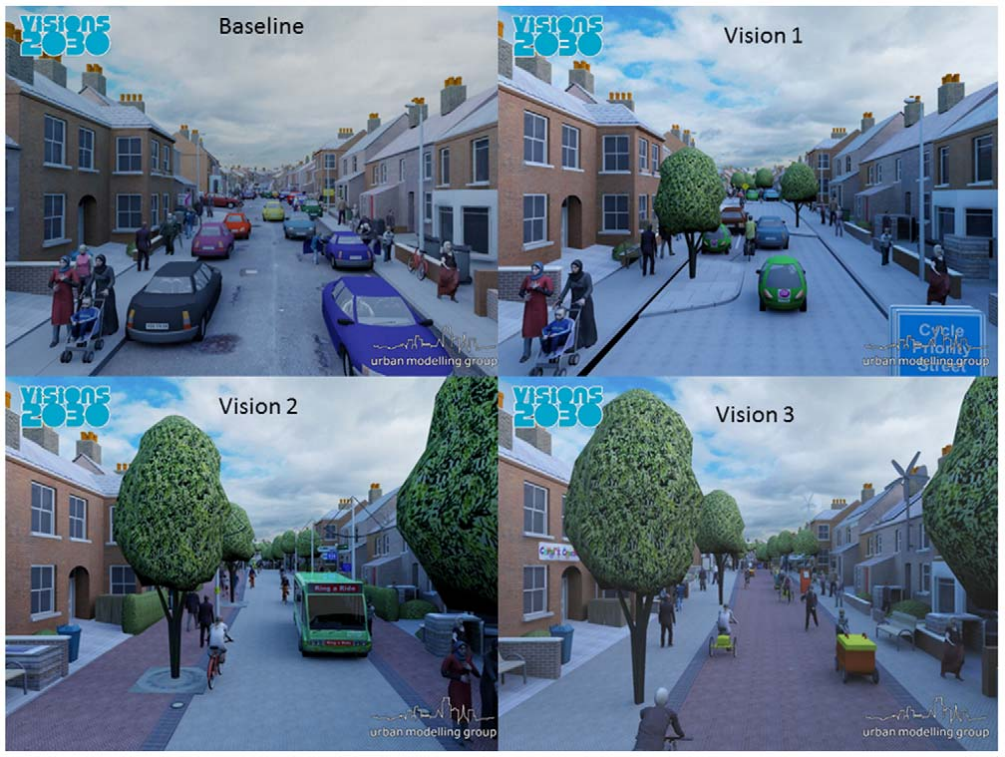
Visualisations for a typical urban terraced street. The four figures are taken from the visualisations used in the Visions 2030 Walking and Cycling Project http://www.visions2030.org.uk/. Each vision represents four different possibilities for urban transport in 2030 in the UK. These visualisations are of a ‘typical’ Victorian terraced street. Visualisations created by the School of Computing at the University of East Anglia.

The three Visions each represent a different image of (or vision for) the UK urban environment in 2030 with substantially higher levels of walking and cycling than is currently the case. Figure 1 shows visualisations corresponding to each of the Visions for an urban terrace street. Complete data for each Vision are shown in Table 3.

**Table 3:** Stages, speed, time, and distance by mode for each Scenario.

		Vision 0		Vision 1		Vision 2		Vision 3	
Stages/week	walk^1^	5.1	25%	6.3	31%	7.4	37%	7.7	39%
	cycle	0.3	2%	2.7	13%	4.3	22%	7.7	39%
	bus	1.1	6%	1.9	9%	3.4	17%	1.9	10%
	minibus	0.2	1%	1.1	5%	2.4	12%	0.5	3%
	train/tube	0.3	1.4%	0.6	3%	1.1	6%	0.6	3%
	car short^2^	8.8	44%	4.1	20%	0.5	3%	0.6	3%
	car long	4.2	21%	3.2	16%	0.5	2%	0.4	2%
	motorbike	0.07	0.3%	0.05	0.2%	0.05	0.3%	0.05	0.3%
	alternative electric vehicle	0.0	0%	0.1	1%	0.4	2%	0.6	3%
	total	20	100%	20	100%	20	100%	20	100%
Mean stage Distance (km)^3^	walk	1.2		1.2		1.3		1.7	
	cycle	3.6		3.6		3.6		4.4	
	bus	11		11		13		14	
	minibus	4		4		4		4	
	train	44		35		35		45	
	car short	3.7		3.7		3.7		3.7	
	car long	31		30		30		28	
	motorbike	16		16		18		20	
	alternative electric vehicle	3.6		3.0		3.2		4.0	
Mean speed (kmph)	walk^4^	4.3		4.6		4.9		5.2	
	cycle^5^	12		13		14		16	
	bus	23		26		23		23	
	minibus	13		13		13		13	
	train^6^	60		45		50		45	
	car short	21		18		15		15	
	car long	51		51		50		35	
	motorbike	42		38		35		30	
	alternative electric vehicle	11		12		13		14	
Distance (km per week)	walk	6.3	3%	8	4%	10	7%	13	11%
	cycle	1.2	1%	10	5.5%	15	12%	34	28.9%
	bus	12	6%	21	12%	43	32%	26	22%
	minibus	1	0%	4	3%	10	7%	2	2%
	train	13	6%	21	12%	39	29%	27	23%
	car short	33	17%	15	9%	2	1%	2	2%
	car long	130	66%	96	55%	14	10%	10	8%
	motorbike	1.1	1%	0.8	0%	0.9	1%	1.0	1%
	alternative electric vehicle	0.1	0%	0.42	0%	1.28	1%	2.40	2%
	total	197	100%	176	100%	133	100%	117	100%
Time (minutes per week)	walk	87	22%	99	24%	118	28%	151	35%
	cycle	6	2%	45	11%	66	16%	127	30%
	bus	32	8%	48	12%	111	27%	67	16%
	minibus	4	1%	20	5%	44	11%	9	2%
	train	13	3%	28	7%	46	11%	36	8%
	car short	96	24%	51	12%	7.5	2%	9	2%
	car long	153	39%	113	28%	16	4%	17	4%
	motorbike	1.5	0.4%	1.3	0.3%	1.5	0.4%	2.0	0.5%
	alternative electric vehicle	0	0%	2	1%	5.9	1%	10	2%
	total	393	100%	407	100%	417	100%	428	100%

1 Walking stages shorter than 250 metres were excluded as they were assumed to be insufficient to contribute to physical activity.

2 Car trips were divided into shorter (<8 km) and longer trips (>8 km). It was assumed that a greater proportion of shorter car trips could be substituted by walking or cycling.

3 Longer stage distances were envisaged for Vision 3 for walking and cycling due to the greater willingness of people to replace longer trips with walking or cycling due to limited availability of motorised transport.

4 Changes in walking speed were assumed to be based on both increased fitness amongst the population and reduced waiting times for pedestrians.

5 Changes in cycling speed were assumed to be based on faster infrastructure and reducing waiting times for cyclists. They were not assumed to affect the intensity of the cycling.

6 Speed for train trips was assumed to fall in Visions 1 and 2 because of greater use of the train for shorter trips. In Vision 3 it was assumed that lower energy availability led to a reduction in speeds even with the longer stage distances.

In the process of creating the Visions, which included an animation of the city in each vision, modal shares were determined and a storyline created to describe the city and allow inference of changes in travel behaviour. Compared with the current situation (Vision 0), in Vision 1 walking and cycling levels are higher (mean minutes per day walking 14.1 and cycling 6.4), assuming a strong trend toward the level currently seen in Dutch cities, while car use is much lower, assuming a trend towards London levels. Socially and economically, Vision 1, is similar to the present day. Vision 2 assumes a far greater emphasis upon social sustainability, with increased egalitarianism, social inclusion and social justice. It includes even higher levels of walking and cycling (mean minutes per day walking 16.8 and cycling 9.5) than Vision 1 and a very low share for car travel (5%), while public transport takes a much larger share of the demand for urban travel than is the case in Vision 0 or 1. Vision 3 occurs in a society that has become very energy conscious due to a widespread shortage of fuel, and in which travel is primarily on foot or by bicycle (mean minutes per day walking 21.6 and cycling 18.2).Compared with Vision 2, there is less use of public transport and increased use of small light electric vehicles with speeds similar to bicycles.

The data for the various Visions were obtained or assumed as follows. For Vision 0, actual data were obtained using the sources named above for the years 2002 to 2008. For Visions 1, data on walking and cycling use (including distance and time travelled) from different European ‘best practice’ settings were used (Netherlands, London, Denmark) as reference to elicit the input into the model. There are currently no cities with levels of walking and cycling as envisaged in Visions 2 and 3 and the input data for the model were obtained through elicitation of expert opinion. In this process, Visions 0 and 1 were used as a reference and combined with the storylines of Visions 2 and 3 to estimate changes in travel behaviour. An iterative set of discussions amongst the research team resulted in input data to the model (e.g. travel distance by mode) that is consistent with the Visions description and mode shares. A full description of the Visions can be found in [5].

With societal and economic changes, for example high fuel prices in Vision 3, substantial changes in modes shares and travel behaviour are expected. For the purpose of modelling the health effects of the transport changes taking place within the Visions the concern is with travel distances, travel times, travel speeds and *who* is doing the travelling. These allow estimation of exposures of physical activity, air pollution and road traffic danger. Societal changes in Visions 2 and 3 might be expected to lead to substantial changes in health exposures and outcomes beyond those directly arising from changes in transport. Even without these changes health outcomes would be anticipated to change by 2030. However, for the purpose of modelling the health effects resulting from changes in the transport mode shares and assumed changes in travel behaviour, only the ‘transport’ effect was considered.

In the scenarios with increased use of typically slower transport modes (primarily walking and to a lesser extent cycling) either travel times have to increase or travel distances have to fall. We modelled a small increase in mean travel times (e.g. from 56 minutes to 61 minutes per day in Vision 3) and a larger fall in travel distances (e.g. 28 km per day to 17 km per day in Vision 3), see Table 3.

Based on the changes occurring in the Visions we also modelled a reduction in freight (heavy goods vehicles and light goods vehicles) distance: a 13% reduction from baseline in Vision 1, 30% in Vision 2, and 39% in Vision 3. In the Visions policies to encourage active transport was assumed to result in the distance freight vehicles travelled on minor roads falling more than the distance on major roads or motorways. Some of the increase in cycling and use of alternative electric vehicles in Visions 2 and 3 would be for final stage freight deliveries [5]. This approach fits with earlier proposals for measures to reduce urban mortality amongst cyclists from heavy goods vehicles [7].

A number of factors were assumed to lead to changes in speeds by mode. Reduced waiting times and better infrastructure were assumed to increase speeds for cyclists and pedestrians. Increased fitness was also assumed to increase walking speeds. For motor vehicles, traffic calming and speed controls on driver behaviour, with automatic speed reduction, were assumed to result in slower traffic speeds and greater conformity to speed limits. Direct effects of changes to congestion on speed were not modelled but might not increase average speeds in a context of road space allocation to pedestrians and cyclists, and other substantial changes to road design.

### Physical activity

A simplified schematic representation of the physical activity model is shown in Figure S1.

For each scenario we used ITHIM to convert mean whole population walking and cycling times to age group and sex specific distributions of metabolic equivalent of task (MET) hours per week [8]. First, we calculated how walking and cycling times vary by age group and by sex by using ratios of walking time and cycling time to a reference age and sex group. For Vision 0, these ratios were derived from the National Travel Survey dataset [9]. To estimate walking and cycling times for people aged 70 to 79 years and 80 years and over we used ratios calculated from the London Travel Demand Survey 2005 to 2008 [10] and applied these to the National Travel Survey age band of 70 years and over. For all other scenarios ratios were informed by those observed in the Netherlands Travel Survey 2005 [11]. The age and sex ratios are shown in Table 4.

**Table 4:** Ratios of time spent walking and cycling compared with women aged 15–29^1^.

		Vision 0^2^		Vision 1^3^		Vision 2		Vision 3	
	Age	m	f	m	f	m	f	m	f
**Walking time**	0–4^4^	1.0	0.9	0.8	0.9	0.7	1.0	0.6	0.6
	5–14	1.0	1.0	0.7	0.8	0.6	0.8	0.6	0.8
	15–29	0.8	*1.0*	0.8	*1.0*	0.7	*1.0*	0.8	*1.0*
	30–44	0.6	0.9	0.8	1.0	0.9	0.9	0.9	0.9
	45–59	0.7	0.8	1.0	1.2	1.0	1.1	1.0	1.1
	60–69	0.8	0.8	1.3	1.3	1.2	1.2	1.1	0.9
	70–79	0.8	0.7	1.3	1.1	1.2	1.0	1.0	0.8
	80+	0.6	0.4	0.9	0.6	0.9	0.6	0.9	0.6
**Cycling time**	0–4^4^	0.5	0.4	0.5	0.4	0.5	1.0	0.7	0.7
	5–14	3.9	1.4	1.1	1.0	1.1	0.8	0.9	0.8
	15–29	4.0	*1.0*	1.1	*1.0*	1.1	*1.0*	1.1	*1.0*
	30–44	3.7	1.3	0.8	0.9	0.8	0.9	1.0	1.0
	45–59	3.4	1.2	0.8	0.9	0.8	1.1	1.0	1.0
	60–69	2.0	0.8	1.0	0.8	1.1	1.0	1.1	1.0
	70–79	1.9	0.3	0.9	0.5	0.9	0.8	0.8	0.7
	80+	0.4	0.2	0.4	0.1	0.4	0.2	0.4	0.4

1 Within each Vision the ratio is that of time spent walking or cycling in each demographic group to that time spent walking or cycling amongst women aged 15–29 (the reference category).

2 Based on data from the UK National Travel Survey 2002–2008.

3 Based on data from the Netherlands National Travel survey 2005.

4 Values for younger children include time being pushed or carried.

**Figure 2:**
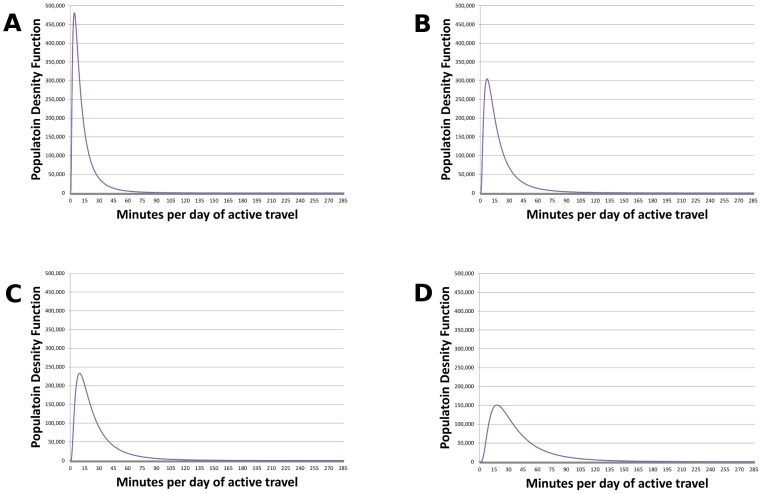
Population distributions of time spent in active travel. A: Vision 1 median 9 minutes per day of walking plus cycling. B: Vision 2 median 14 minutes per day of walking plus cycling. C: Vision 3 median 19 minutes per day of walking plus cycling. D: Vision 4 median 30 minutes per day of walking plus cycling.

We then combined walking and cycling time to give total active travel time. As time spent walking and cycling is skewed to the right, we fitted log normal distributions for each demographic group. The standard deviation was specified to represent the weekly active travel distribution. The coefficient of variation for each distribution was assumed to fall as average time spent in active travel increased. This was based on an algorithm we developed from analysis of multiple travel surveys (UK, London and the Netherlands), see below. The distributions of active travel time for the whole population under each Vision are shown in Figure 2, converted into equivalent minutes per day.
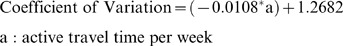



Walking and cycling are different intensity activities [8]. It was assumed that all cycling had an intensity of 6.8 METs (“bicycling 10–11.9 mph leisure, slow, light effort”, “bicycling, to/from work, self-selected pace”).[12] For walking we created an algorithm to convert mean walking speed for each demographic group to MET values based on published data, assuming a minimum intensity of 2.5 METs (slow walking) [12]. The walking and cycling active travel time distribution was then converted into a MET hours distribution based on these calculated values.
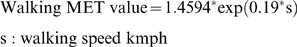



Non-travel related physical activity was estimated using the Health Survey for England (HSE) for 2008. It was assumed that these activities would not change across the scenarios. For each quintile of walking and cycling activity in HSE within each demographic group we estimated weekly MET values for activity from all the other domains (see Table S1). These quintile specific median MET values were added to the active travel quintile specific median MET values to estimate total physical activity exposure.

In Vision 3, in which there is an uptake of small light electric vehicles (including electric bikes and mobility scooters), simplifying assumptions were made that these were not physically active modes and that the risk of injury was the same as for pedal cycles.

#### Modelling health impacts

In the health impact model median MET hours per week of active travel or total physical activity for each quintile of the MET hours distribution were used as the measures of physical activity exposure. Separate distributions were used for each age and sex group. The change in exposure was the change in median MET hours for each quintile of the distribution comparing each Vision with baseline. How the age and sex specific disease burdens would change under each scenario was then modelled using a comparative risk assessment approach [13].

The impact of physical activity on different diseases was based on the systematic overview in Woodcock et al 2009 [2], (Table 5). For type II diabetes [14] and cardiovascular disease [15], relative risks were taken from systematic reviews based on walking alone, therefore, only active travel exposure was considered. For breast cancer [16], colon cancer [17], dementia [18], and depression [19] relative risks were taken from systematic reviews and other studies combining physical activity from multiple domains. It was assumed the same relative risks applied to premature deaths, years of life lost, and years of healthy life lost due to disability. Studies suggest that the relationship between physical activity and health outcomes is strongly curvilinear, with the greatest benefit from moving from low to moderate levels of activity [20], [21]. However, the shape of the relationship for different diseases is uncertain. Given the evidence of a curvilinear relationship in general but an absence of evidence on the shape of the relationship for specific diseases it was assumed, in the main analysis, that changes in disease risk were log linearly associated with a power 0.5 transformation of the exposure.

**Table 5:** Dose response functions used by disease and for all-cause mortality.

	Specific diseases for main model^1^								All-cause mortality				Sensitivity analysis for cardiovascular disease		
	Breast Cancer [16]	Colon cancer [17]		Cardiovascular disease [15]	Dementia [18]	Depression [19]		Diabetes [14]	Woodcock et al [20] total activity	Woodcock et al [20] walking alone	HEAT for cycling^2^ [23]	HEAT for walking^3^ [24]	Sattelmair [21]		
Population restrictions	female	male	female			Age>29	Age 15–29								
Exposure (MET hours) corresponding to relative risk	5	31	30	8	32			11	11	11	18	12	11	23	56
Relative Risk^4^	0.94	0.80	0.86	0.84	0.72	0.83	0.93	0.89	0.81	0.89	0.72	0.78	0.86	0.80	0.75
Transformation of exposure	0.5	0.5	0.5	0.5	0.5	0.5	0.5	0.375	0.25	0.375	No transformation	No transformation	No transformation within each spline		

1 . Methods for estimating exposures and selection of relative risks are reported in Woodcock et al 2009 [2]. Transformation of exposure for specific diseases is reported here for the main model (alternative transformations 0.25, 0.375, log, and 1 were used in sensitivity analysis.

2 . 0.72 for 3 hours per week of cycling at 14 km/hour.

3 . 0.78 29 minutes per day of walking at 4.8 km/hour.

4 . Reduction in health outcome as a change in walking or cycling = 1 – RR ∧ (a/b).

a = Scenario MET hours per weeks∧ Transformation of exposure.

b = Reference MET hours per week ∧ Transformation of exposure.

The log normal distribution approach for modelling physical activity assumes everyone undertakes some active travel. However, in the cohort studies used to estimate dose response relationships between physical activity and health outcomes people doing less than a certain amount of physical activity would be classified as inactive. Therefore, a minimum threshold of 2.5 MET hours per week was assumed for any benefit from physical activity (this is equivalent to an average daily total of fewer than 10 minutes per day of slow walking).

We used disease burden data from the WHO for the UK for 2010, reweighted for the size and demographic structure of the English and Welsh urban population outside London [22]. We used data that were not age weighted or discounted.

#### Sensitivity analysis

To investigate the sensitivity of the analysis to changes in the parameters and model structure we ran multiple sensitivity analyses.

We considered alternative dose response functions based on alternative transformations of the exposure (no transformation, log, power 0.375, and power 0.25) and using values from a separate meta-analysis of physical activity and ischemic heart disease, which fitted cubic splines, (Table 5) [21].

We directly modelled the impact of changes to physical activity exposure on all-cause mortality rather than modelling disease specific mortality. Exposure response functions for all-cause mortality taken from Woodcock 2010 [20] were used for total physical activity and for walking alone, (Table 5).

The World Health Organization HEAT tools for walking and cycling model changes in premature deaths from changes to walking and cycling, respectively [23] [24]. They use a log-linear model, i.e. the exposure variable is not transformed. Additionally, the HEAT tools are only intended to be applied to populations aged under 75 years for walking and under 65 years for cycling, whereas the ITHIM tool models impacts from changes in physical activity to all age groups over 30 years old. We ran multiple comparisons with the HEAT tools and the values they provided.

Firstly, using ITHIM, we applied the exposure response functions recommended for the WHO HEAT tools for cycling [23] and for walking [24] (Table 5). The relative risks were then separately applied to the change in total active travel (i.e. walking and cycling combined). Next, we ran models with and without the age restrictions – that is, assuming no effect on those aged >74 years when using the relative risks for walking or >64 years when using the relative risks for cycling. To facilitate comparability with the main results from ITHIM we modelled the impact on both premature deaths and years of life lost, but not on years of healthy life lost due to disability.

Secondly, the HEAT tools for cycling and walking were directly used to estimate the percentage reductions in deaths due to changes in cycling activity and walking activity, respectively. We report both the results assuming an effect on the whole population as well as adjusted results. These adjusted results were estimated by calculating the proportion of all deaths in our study population occurring amongst people aged under 75 (for walking) and 65 (for cycling), and multiplying this proportion by the percentage changes in total deaths. We combined the impact fractions from the HEAT walking model and the HEAT cycling model assuming a multiplicative rather than additive relationship to reduce the risk of double counting.

The ratio of time spent walking and cycling by different age groups was assumed to differ between the Visions, becoming more in line with that of the Netherlands as cycling increases (Table 4). To investigate the sensitivity of the model to these changes, we modelled the impact of changes in total population activity levels assuming that age and gender ratios remained constant.

### Injury risk

In this study we used a risk, distance and speed based modelling approach. Changes in the distance travelled by motor vehicle traffic and by pedestrians and cyclists in the transportation scenarios are likely to affect the number of road traffic injuries and who is injured. We developed a model to generate absolute numbers, rather than relative risks, of deaths and serious injuries from road traffic collisions. A simplified schematic of the road traffic injury model is shown in Figure S2.

We treated all injuries as being either the result of a collision between a striking vehicle and a victim vehicle occupant (or pedestrian) or being the result of a collision involving no other vehicle. For collisions involving multiple vehicles we categorised the largest vehicle as the striking vehicle. The model is based on changes to distance travelled by striking vehicle modes, changes to distance travelled by victim mode and on changes to motor vehicle speed. Changes to distance are applied to the numbers of injuries suffered by users of a given mode involved in a collision with another given mode. A related modelling approach was proposed by Bhalla et al [25] in which a two stage process was used to calculate collisions and then injuries. The current study used a one stage approach to directly calculate injuries and fatalities. This model is closely related to that applied previously for California [6] and London [2] but different parameters were used in this current study.

We analysed routinely collected UK Police data from the Stats19 database [26] from 2002–2008 to separately estimate the number of victims seriously injured and killed in collisions with each type of vehicle. It was assumed that injury risks would differ on major and minor roads. Therefore, separate tables of injuries were extracted from Stats19 for injuries on each of the road types; motorway, A road, and minor road. Where collisions occurred at a junction between two road types, the higher road classification was used. Collisions were geocoded using ArcGis and we selected injuries that occurred in urban areas in England and Wales outside London. The Stats19 classification of injuries (fatal, severe and minor) was used. A ‘fatal’ injury is defined as those cases in which death occurs in fewer than 30 days as a result of the collision. ‘Serious’ injuries are following hospital admission an in-patient either immediately or later. Minor injuries were not considered due to reporting inaccuracies [27]. To illustrate the structure of the data, values for serious injuries on minor roads are shown in Table S2.

We modelled changes in the number of people injured in collisions involving each combination of modes based on changes in person distance (for victims) and vehicle distance (for striking vehicles). Person distance and vehicle distance are related through mode specific occupancy rates. We took empirical estimates provided by Elvik [28] that suggest increases in distance either by striking vehicle or by victim mode lead to a less than proportional increase in injuries. The formulae are presented below.
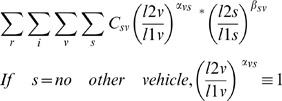

- r = road type (motorway, major, minor)
- i = injury type (fatal, serious)
- v = victim mode (pedestrian, cyclist, motorcyclist, car occupant, LGV occupant, bus occupant, HGV occupant)
- s = striking mode (bike, motorbike, car, LGV, bus, HGV, no other vehicle)
- C = casualties for a given victim mode and striking vehicle at baseline
- l2 = distance by mode in scenario
- l1 = distance by mode at baseline
- If victim mode is cyclist or pedestrian then βsv = 0.7 & αvs = 0.5 (cyclist) or 0.4 (pedestrian)
- If victim mode is one of (motorcyclist, car occupant, LGV occupant, bus occupant, HGV occupant) then βsv and αvs = 0.525
- If striking vehicle is no other vehicle then αvs = 0.8

These values are such that for collisions involving a motor vehicle with a bike or pedestrian, a doubling of motor vehicle distance leads to a 62% increase in injuries amongst cyclists and pedestrians, while for victims a doubling of distance by cyclists leads to a 41% increase in cyclist injuries and a doubling of distance by pedestrians leads to a 32% increase in pedestrians injuries. For collisions solely involving motor vehicles, a doubling of distance by striking vehicle or victim vehicle would lead to a 44% increase in injuries (2∧0.525). For the risk of collisions in which the striking vehicle and victim mode are the same then, automatically, a doubling of distance by victim mode would imply a doubling of distance victim by striking mode. In this case, a doubling of distance would lead to an increase in the number of injuries of 107% ((2∧0.525)∧2). For collisions involving only one vehicle, a doubling of distance would lead to a 74% increase in injuries.

Vehicle speed is a strong risk factor for collisions and severity of injury. Therefore, we adapted a simple version of the speed model described by Cameron & Elvik [29] to model how changes to speed would affect injuries. In this modelling method, the relationship between speed and injury risk is strongly non-linear and is based on different power transformations for different road types. To represent changes to speeds on these roads it was assumed that for each Vision the specified change in average car speed for shorter car trips equated to the change in average speed on minor roads, and the change in average car speed for longer car trips equated to the change in average speed on major roads and motorways. It was also assumed that the speed model could only be robustly applied to collisions in which cars, light goods vehicles or motorbikes were the striking vehicle.
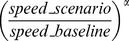
α = 3.5 for motorways and 2 for major and minor roads

Changes in the demographic structure of travel by different modes may affect the changes in disease burden as loss of life in an older age group is calculated as fewer years of life lost than a death at a younger age. The simplifying assumption was made that the total numbers of injuries by mode were not affected by changes in who was cycling or walking but that the modelled change in injuries among cyclists and pedestrians was distributed across demographic groups according to the change in time spent walking and cycling in each demographic group. The change in disease burden was measured as DALYs, years of life lost, and years of healthy life lost to disability. To calculate this from changes to numbers of serious injuries and fatalities, we assumed the ratios of deaths to years of life lost due to deaths was a constant within each demographic group based on the data provided in the WHO disease burden data, and the ratio of years of life lost to years of healthy life lost due to disability to injuries was a constant within each demographic group.

#### Sensitivity analysis

We undertook sensitivity analyses varying the relationship between the changes in distance for each mode and changes in number of injuries. Firstly we assumed that injuries changed with the square root of the ratio of distance in the scenario to distance at baseline for both victim mode and striking vehicle (as used in the California application of ITHIM [6]) and secondly we assumed a simple linear relationship between injury and distance (as used in the modelling for London and Delhi [2]). We also ran the model excluding the speed module.

### Air pollution

A simple model was used to estimate the health impacts from changes to air pollution. We used published data that provides concentrations of particulate matter smaller than 2.5 micrometres (PM 2.5) and source apportionment (for primary PM) for each 1 kilometre (km) square grid in the UK [30]. Combining this data with data from the 2001 census we calculated population weighted PM 2.5 for urban areas in England and Wales outside London [31]. These data were weighted by age group specific population data (age groups: 0 to 4, 30 to 59, and 60+). We then took data on exhaust pipe and tyre wear emissions per km for each vehicle type for urban areas by road type for 2008 from the UK National Air pollution Emissions Inventory; see Table S3 [32].

The distance travelled under each scenario for each vehicle type was multiplied by the emission factors for that mode to produce estimates of the change in total transport emissions. The assumption was made that emission factors were constant across all the Visions. Finally we assumed that a change in urban transport emissions translated into a proportional change in primary PM 2.5 attributable to urban transport. The effect of changes in traffic congestion on air pollution was not modelled. A simplified schematic of the air pollution model is shown in Figure S3.

To calculate health impacts from changes to PM exposure we used the values recommended by the WHO [33], (Table 6). Changes to deaths and years of life lost were modelled across all cardio-respiratory diseases, lung cancer, and for acute respiratory infections in children aged less than 5 years.

**Table 6:** Air pollution health impact model.

	Model	Coefficient
Cardio-respiratory (age> = 30)	RR = exp(b(×1–×2)	b = 0.00893
Lung cancer (age> = 30)	RR = exp(b(×1–×2)	b = 0.01267
Acute respiratory infections (age<5)	RR = exp(b(×1–×2)	b = 0.00332

×1 = exposure baseline.

×2 = exposure scenario.

GBD cause groups. Cardio-respiratory 39–40, 106–109, 112–114; Lung cancer 67; ARI 39–40.

Both air pollution and physical activity affect the risk of cardiovascular diseases and so to reduce the risk of double counting we assumed that percentage reductions in the disease burden were multiplicative rather than additive.

#### Sensitivity analysis

A second, simpler approach was used for sensitivity analysis that aimed to capture in broad terms changes in secondary PM. In this model, we took national values for the proportion of PM 2.5 emissions, including from natural sources, due to road transport and assumed a proportional reduction in total concentrations (based on the weighted average for the urban population of England and Wales) equivalent to the percentage reduction in emissions [34].

### Carbon dioxide

For carbon dioxide (CO_2_) emissions, we used mode specific emission factors from the National Atmospheric Emissions Inventory 2008 [32]. The assumption made was that emission factors were constant across all the scenarios (see Table S4). Based on the changes in vehicle km by road type we modelled the change in CO_2_ emissions for vehicle distance by passenger transport, both private motor vehicles and buses.

### Software for data analyses

Data analyses were conducted in Stata 11 (STATA Corporation) and ArcGIS (Esri). Health impact modelling was conducted in Excel 2010 (Microsoft), using ITHIM.

## Results

**Figure 3:**
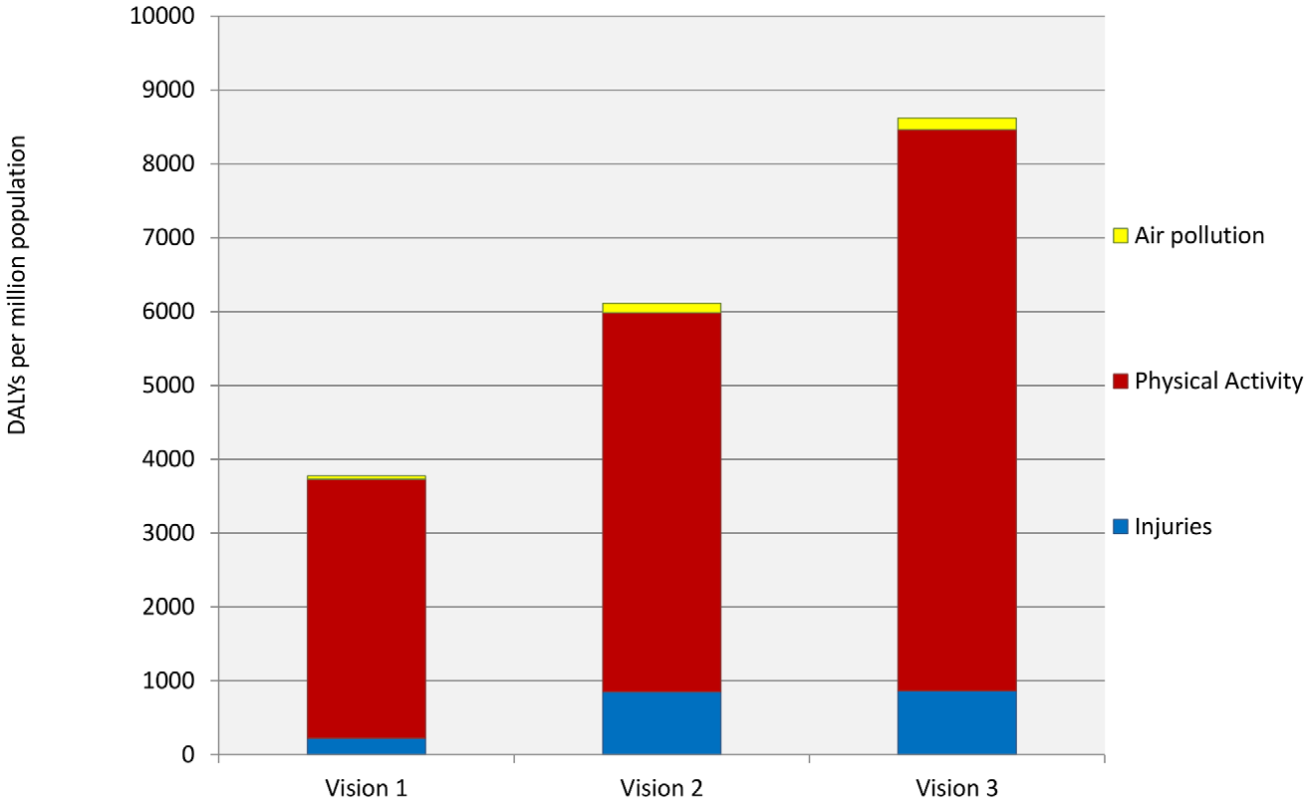
Health gains by Vision and risk factor. Disability Adjusted Life Years gained per million population under each of the three visions, broken down into the proportions attributable to improvements from air quality, increased physical activity and decreased road injuries. See Table 7 for full results.

Results for each scenario standardised as DALYs per million population corresponding to the change in disease incidence in one year are show in Table 7 and Figure 3. In all scenarios, there was a reduction in the disease burden with the largest contribution coming from increases in physical activity, followed by fewer road traffic injuries. The largest health gains were from changes in ischemic heart disease, stroke, and dementia, followed by reductions in injuries (Visions 2 and 3) or diabetes (Vision 1); full data are shown in Table 8. The percentage reduction in different diseases affected by physical activity varied according to 1) the strength of the relationship between physical activity and the health outcome, 2) the changes in exposure in different demographic groups.

**Table 7:** DALYs gained in one accounting year by Vision and by risk factor per million population.

	Vision 1	Vision 2	Vision 3
Physical activity	3503	5129	7595
Air pollution	47	137	166
Road traffic injuries	228	855	867
Total	3774	6106	8606
Reduction in total disease burden^1^	1.8%	2.9%	4.1%
Reduction in CO_2_ emissions from passenger transport by urban residents	26%	73%	83%

1 . Results do not total due to adjustment for double accounting.

**Table 8:** Health gains by disease category.

Vision		Ischemic heart disease	Stroke	Dementia	Injuries	Diabetes	Depression	Breast cancer	Colon cancer	Hypertensive heart disease	Lung cancer	Respiratory diseases	Inflammatory heart disease
1	DALYs per million population	−1470	−758	−610	−228	−310	−154	−80	−99	−48	−10	−7	−1
	% of disease burden	−7.6%	−7.0%	−5.3%	−15.6%	−7.2%	−2.0%	−1.8%	−2.2%	−7.1%	−0.2%	0.0%	−0.1%
2	DALYs per million population	−2089	−1120	−954	−855	−439	−248	−120	−161	−70	−28	−19	−2
	% of disease burden	−10.8%	−10.3%	−8.3%	−58.5%	−10.2%	−3.3%	−2.7%	−3.6%	−10.4%	−0.4%	−0.1%	−0.2%
3	DALYs per million population	−2998	−1637	−1432	−867	−629	−451	−183	−246	−103	−34	−23	−3
	% of disease burden	−15.6%	−15.1%	−12.5%	−59.3%	−14.6%	−5.9%	−4.2%	−5.5%	−15.2%	−0.5%	−0.1%	−0.3%

Overall this resulted in a reduction in the total population disease burden of up to 4.1% with Vision 3. To put these figures into the context of more general values, the WHO estimates that for high income countries as a group lack of physical activity is responsible for 7.7% of disease burden (using different relative risks from those used in this study), with overweight and obesity an additional 8.4% and tobacco 17.9% [35].

### Road traffic injuries

We found a reduction in the disease burden from road traffic injuries (DALYs) in all scenarios: 16% in Vision 1, 58% in Vision 2, and 59% in Vision 3. The changes in fatalities and serious injuries by mode are given in Tables 9 and 10 respectively.

**Table 9:** Fatalities by mode per scenario.

	Fatalities^1^			
	Baseline	Vision 1	Vision 2	Vision 3
walk	380	315	215	168
cycle & alternative electric vehicle	53	133	129	177
bus	11	18	32	20
car	424	267	55	28
HGV	21	21	19	16
LGV	16	15	12	7
motorbike	121	71	51	32
total	1025	839	514	447

**Table 10:** Serious injuries by mode per scenario.

	Serious injuries^1^			
	Baseline	Vision 1	Vision 2	Vision 3
walk	4380	3596	1847	1636
cycle & alternative electric vehicle	1449	3244	2040	2955
bus	222	390	741	425
car	4968	3168	537	296
HGV	130	124	108	96
LGV	177	162	124	73
motorbike	2235	1378	777	558
total	13561	12062	6175	6039

1 . Percentage reductions in these tables differ from the change in disease burden due to the different loss of life expectancy with a death or injury at different ages.

In Vision 1 we found an increase in injuries amongst women aged over 45 and in men aged over 60 (figures not shown). However, these increases were outweighed by the other health gains from increases in physical activity for both of these age groups, suggesting a net benefit in all age groups.

### Air pollution

Particulate matter from transport was reduced by approximately two thirds in Vision 3 and total exposure to particulate matter was reduced by 0.1 µm in Vision 1, 0.4 µm in Vision 2, and 0.5 µm in Vision 3. Full results are shown in Table 11. Changes to air pollution had a smaller impact on total health outcomes than change in road traffic injuries and a much smaller impact than changes in physical activity (DALYs per million population: Vision 1, 47 DALYs; Vision 2, 137 DALYs; Vision 3 166 DALYs; see Table 7).

**Table 11:** PM 2.5 Values by Vision.

	Baseline	Vision 1	Vision 2	Vision 3
Population weighted PM 2.5 exposure (µm) 30–59 year age group	10.3	10.2	9.9	9.8
Sensitivity analyses: Population weighted PM 2.5 exposure (µm) 30–59 year age group	10.3	9.9	9.1	8.9
Reduction in emissions from urban transport	0%	19%	51%	69%
% PM 2.5 from local road transport	17%	14%	8%	6%

### Sensitivity Analyses

#### Physical activity

Changes to the shape of the dose response relationship had a large impact on the results. A log linear model without a transformation of the exposure found gains that were in some cases more than double that of a power 0.5 relationship. Smaller effects were found (in order) from a log transformation of the exposure, a power 0.375 transformation, and power 0.25 transformation (see Table 12).

**Table 12:** Impact of physical activity dose response relationship on reduction in disease burden from ischemic heart disease.

Percentage reductions IHD	Power transformation of exposure					*based on different RRs
	0.25	0.375	log	0.5	linear	Cubic splines*
Vision 1	5.1%	6.5%	7.7%	7.6%	12.3%	4.0%
Vision 2	6.8%	8.9%	10.3%	10.8%	19.7%	6.3%
Vision 3	8.9%	12.2%	13.4%	15.6%	32.7%	9.5%

When relative risks were used for the effect of physical activity directly on all-cause mortality a larger impact was found on years of life lost than from combining changes from individual diseases, see Table 13. The reduction in all-cause mortality were similar using the relative risk for walking (applied to changes in active travel) and the relative risk for total physical activity (applied to changes in total activity).

**Table 13:** Percentage reduction in years of life lost (YLLs) and premature deaths using ITHIM and HEAT.

		ITHIM								HEAT tool				
		ITHIM main model		ITHIM all-cause mortality		ITHIM using HEAT relative risks								
		Main model power 0.5 dose response	Main model power 1 dose response	All-cause mortality using RRs for walking from Woodcock 2010 (20)	All-cause mortality using RRs for total physical activity from Woodcock 2010 (20)	ITHIM using HEAT walking RRs (without age exclusions)	ITHIM using HEAT walking RRs (with age exclusions)	ITHIM using HEAT cycling RRs (without age exclusions)	ITHIM using HEAT cycling RRs (with age exclusions)	HEAT tool walking (as % of deaths in people aged 20–74)^1^	HEAT tool walking (as % ofall deaths)	HEAT cycling (as % of deaths in people aged 20–64)^2^	HEAT tool cycling (as % of all deaths)	HEAT walking and cycling combined (as % of all deaths)^3^
YLLs	Vision 1	2.0%	3.2%	3.3%	3.6%	10.3%	7.9%	9.3%	4.2%					
	Vision 2	3.0%	5.1%	4.7%	4.9%	14.5%	12.7%	15.2%	6.9%					
	Vision 3	4.3%	8.9%	6.7%	6.9%	28.8%	21.8%	26.2%	12.9%					
Deaths	Vision 1	2.2%	3.2%	4.3%	3.4%	8.8%	6.6%	7.9%	1.8%	2.1%	0.8%	10.0%	1.9%	2.7%
	Vision 2	3.3%	5.2%	6.1%	4.9%	14.3%	9.7%	13.1%	2.8%	4.3%	1.7%	15.2%	2.9%	4.6%
	Vision 3	5.0%	8.7%	8.7%	7.1%	25.1%	14.7%	22.9%	5.1%	8.1%	3.2%	29.0%	5.6%	8.5%

1 . These results are from changes to walking exposure alone. These are directly taken from HEAT Walking, comparing the results for mean baseline walking time versus the results for mean walking time under each Vision. They represent percentage reductions in disease burden in people aged <74 years.

2 . These results are from changes to cycling exposure alone. These are directly taken from HEAT Cycling, comparing the results for mean baseline cycling time versus the results for mean cycling time under each Vision. They represent percentage reductions in disease burden in people aged <65 years.

3 . The change in deaths/YLLs amongst the affected population (age <65 years walking, <75 years cycling) as a percentage of deaths in the whole population.

Results from the models using the HEAT tools' relative risks generally produced larger health impacts than using ITHIM relative risks. This was the case even when applying non-transformed dose response curves in ITHIM, indicating that both the relative risk point estimate and the shape of the exposure response curve within HEAT contribute to the larger result.

Using HEAT directly produced an even larger result in terms of proportion of deaths for given age groups than that from using the HEAT relative risks within ITHIM (see Table 13). However, applying the HEAT guidance by excluding the impact of physical activity on mortality amongst older adults considerably reduced the proportion of premature deaths averted in the whole population, because of the high number of deaths in older people. At an older age a death contributes less to years of life lost so the difference in years of life lost was smaller than the difference in deaths. Because HEAT recommends a lower age threshold for cycling than for walking the difference in results are greater for cycling than for walking.

To summarise the comparison, if using both tools with the recommended values (and combining results from changes to cycling and walking in HEAT) there were higher numbers of premature deaths averted with HEAT than with ITHIM if summing individual diseases in ITHIM but typically a smaller number of deaths averted with HEAT than with ITHIM if directly modelling all-cause mortality with ITHIM. However, due to the exclusion of impacts on older age groups with HEAT the premature deaths averted would be at an older average age in ITHIM compared with HEAT, and hence would tend to correspond to fewer years of life lost.

If it was assumed that age and sex relative times spent walking and cycling remained unchanged at baseline levels in all scenarios then the absolute health benefit was notably smaller for all the Visions (DALYs: Vision 1, 1.1% vs 1.8%; Vision 2, 2.2% vs 2.9%; Vision 3, 3.3% vs 4.1%).

#### Injury

The sensitivity analysis assuming a power 0.5 found smaller benefits for Visions 1 and 2 but the injury disease burden still fell under all scenarios (DALYs: Vision 1, −13%; Vision 2, −56%; Vision 3, −59%). If a fully linear model was used then the injury disease burden increased under all scenarios, with a large increase in Vision 3 (DALYs: Vision 1, 22%; Vision 2, 4%; Vision 3, 168%).

When the effect of changes in speed was excluded from the mode, the injury burden still fell in all scenarios but by smaller amounts (DALYs: Vision 1; −4%, Vision 2; −51%, Vision 3; −45%).

#### Air pollution

Using the proportion of total emissions PM 2.5 attributable to road transport (20.7%) gave a considerably greater reduction in concentrations and in disease burden for all scenarios. Average exposure was reduced by 0.4 µm in Vision 1, 1.2 µm in Vision 2, and 1.4 µm in Vision 3, with a reduction in disease burden (DALYs per million population) in Vision 1 of 148 DALYs, Vision 2 of 429 DALYs, and Vision 3 of 520 DALYs. However, these reductions are still smaller than those from changes in injury and much smaller than those from changes in physical activity.

#### Carbon dioxide

In Vision 1, CO_2_ emissions from passenger transport by people who live in urban areas fell by 16 megatonnes (Mt) (26%), by 44 Mt in Vision 2 (73%) and by 50 Mt in Vision 3 (83%) (Table 14).

**Table 14:** Megatonnes of CO2 from people living in urban areas by Vision^1^.

	Vision 0	Vision 1	Vision 2	Vision 3
Passenger cars, taxis, motorbikes & mopeds	57.6	39.6	5.6	4.4
Buses	2.7	5.2	10.6	5.6
Total	60.3	44.7	16.2	10.1

1 . Additional reductions in emissions due to changes in freight were not modelled, nor were increases in emissions due to rail.

## Discussion

### Principal findings

In this study we found that a shift to a more physically active and less car based transportation system could provide important benefits for population health by increasing physical activity, reducing road traffic injuries, and reducing air pollution; while also reducing greenhouse gas emissions. Sensitivity analyses highlight the importance of increasing walking and cycling amongst older age groups.

### Strengths and limitations of the study

This study is the first study to model scenarios for England and Wales outside of London. A previous health service economic modelling study took results from London and extrapolated these to the rest of the England and Wales but did not model detailed new scenarios for England and Wales [36]. There are a number of strengths to the current study. It is based on the use of a health impact modelling tool, combining transport analysis with health, injury risk and pollution analyses. The study drew on large datasets covering multiple years to provide information on travel patterns by age and gender and on non-travel physical activity. A further strength is the use of a more sophisticated physical activity and road traffic injury model than that used in earlier studies, including integration of the impacts of speed on road traffic injuries. The multiple sensitivity analyses and comparisons with the main other modelling tools, the HEAT tools, provide information on the extent to which different parameters and modelling assumptions lead to different results.

The study also has a number of limitations. The use of a simple air pollution model only considering the effects of PM 2.5 and not including a dispersion model may mean the benefits from reductions in air pollution are underestimated [37]. Alternatively, the results may be overestimated as we did not model potential effects of higher ventilation rates of pedestrians and cyclists compared with motor vehicle occupants [38]. Another limitation is due to the comparative risk assessment method used for physical activity and air pollution. This method is only able to estimate committed gains between two static comparisons and cannot reliably estimate changes over time. The change in injuries over time was also not modelled.

The household travel surveys and the Health Survey for England suffer from the limitations of self-reported data. In the travel surveys shorter walking trips in particular may be underreported. Reporting accuracy over the week is likely to fall off. Walking and cycling away from public roads is inadequately captured in the surveys. Stats19 is police reported data, although it is likely to be accurate for fatalities, it will miss some serious injuries, and many minor injuries [27]. For this reason we did not model changes to minor injuries. Injuries not involving a motor vehicle are particularly likely to be underreported for cycling and falls or other injuries sustained while walking, that do not result from an impact with a motor vehicle, are not recorded.

When modelling physical activity and air pollution, no lagged impact on older age groups was modelled; that is, a change in exposure amongst one age group was assumed to lead to changes in health outcomes for that age group alone. Support for most of the impact from changing behaviour occurring in less than 10 years is provided by Byberg et al 2009 [39]. The physical activity model uses relative risks based on physical activity from multiple domains for most diseases, but on walking alone for diabetes and cardiovascular disease. If non-transport physical activity is similar between the populations in which the walking studies were conducted and the population to which the modelling results are applied, then the use of walking specific relative risks would be appropriate. The similarity of the impact on all-cause mortality using either the walking relative risks combined with active travel physical activity alone or with the results using all activity relative risks combined with total physical activity encourages confidence in the use of these estimates. Only some health impacts were included in the model. This study did not model an effect on overweight and obesity so the total benefits may be greater than we currently identified. Other health pathways, such as noise pollution were also not included.

### Comparison against other studies and models

Earlier studies for London [2], the Netherlands [40], Barcelona [41], New Zealand [42], Copenhagen [43]m and California [6] have found increases in road traffic injuries with climate change mitigation active travel scenarios. It is therefore encouraging that we found a reduction in road traffic injuries in a high income country. The finding of a reduction in injury risk may appear counterintuitive. This finding is likely to be related to the following factors:

1. The inclusion of changes to travel by ‘striking vehicle’ in addition to the mode of the injured person combined with the very large reductions in motor vehicle distance in Visions 2 and 3.
2. The inclusion of non-linearity of risk with distance factors.
3. The reductions in total travel distances in the more active scenario.
4. The inclusion of a speed model.
5. The inclusion of changes to freight in the scenarios.

The main findings of reduction in disease burden for ischemic heart disease (10.8% Vision 2 and 15.6% Vision 3) were smaller than under the main scenarios for London in previous work, which found a 18% reduction using a model based on an untransformed physical activity exposure with a threshold [2]. The average amount of walking and cycling in the previous study's scenarios (cycling 3.4 km, walking 1.6 km per person per day) were lower than those in Vision 3 but higher than those in Vision 2 (see Table 3).

The HEAT tools are the most widely used model for estimating changes in health outcomes following a change in walking and cycling. Some studies have used the HEAT tools directly [37], [44], while other tools have taken various parameters from the HEAT tools and used them as part of other models [3], [40], [41]. It is also used as part of the UK Department for Transport WebTAG guidance [45]. The HEAT tool estimates social benefits by applying a monetised value of a statistical life to the number of premature deaths averted. These tools are simple to use and the methods are transparent. The relative risks for these models and the recommended shape of the dose response relationship produced considerably larger results than those recommended with ITHIM, although the overall impact was offset by the exclusion of effects on older age groups. In HEAT the relative risks for cycling are taken from one study of cycling from Denmark [23]. Because of the small number of cohort studies investigating the effect of cycling on mortality a meta-analysis of cycling studies would not be satisfactory. However, it should be noted that while one other study found a similar large result (albeit with wide confidence intervals) [46] a third study showed no evidence of an effect [47]. Given the limitations of cycling specific evidence and without a strong rationale that cycling provides additional benefits beyond those expected from its MET intensity it would seem reasonable to use a relative risk from other activities of similar intensity.

If the relationship between physical activity and health outcomes is strongly curvilinear, as suggested by reviews [20], [21], [48], then one would expect a smaller effect from a measure of any one type of activity alone, such as cycling, than from a measure of all activities combined. In this case, if a relative risk based on multiple domains of physical activity is used, it is important to estimate the levels of non-cycling activity amongst the population. If a relative risk for walking or cycling specifically is used then it is not necessary to estimate other physical activity given the following two conditions: non-transport physical activity is similar at baseline in the target population to that of the populations in the studies from which the relative risks were derived; and non-transport activity does not change with a change in active travel.

The evidence for walking is considerably greater than that for cycling. The HEAT tool bases its relative risk on a meta-analysis that has not yet been fully published [49]. The results from those meta-analyses for which more detail is available suggest a smaller result [20], [21]. Despite this it should be noted that systematic reviews identify considerable heterogeneity and the confidence intervals only represent some of the uncertainty [15], [50], [51]. It should also be noted that most of the large studies of self-reported physical activity and health outcomes only measured exposure at one point in time and misclassification of exposure over time would lead to underestimation of the results. This would suggest that the relative risks used by ITHIM could be underestimates.

In our scenarios the exclusion of health benefits amongst older people by HEAT leads to a very large reduction in the size of the expected benefits. In some settings, achieving increases in cycling amongst older people may not be seen as feasible in the short term and the exclusion may be appropriate. However, the evidence from the Netherlands indicates that cycling amongst older people is a realistic goal. Relative risks for older age groups may be expected to differ from those for younger groups; however, the existing evidence suggests a larger benefit from low amounts of activity than is the case amongst younger people [20]. Explicit specification of *who* is changing behaviour in health impact modelling broken down by age offers advantages over more aggregate modelling approaches where older age groups are excluded.

### Policy implications

Walking and cycling can be important, everyday, modes of transport if our cities are designed for them. Lower average travel speeds, with a prioritisation of local accessibility over longer distance mobility increase the attractiveness of walking and cycling. These changes would have positive implications for injuries and emissions, with significant health benefits, principally from increased physical activity, that are currently not accounted for when investments in transport (and in cities more generally) are evaluated.

This study is based on scenarios in which there is a step-change in active travel. In the Visions 2030 study Visions 2 and 3 are assumed to occur in the context of substantive social and economic changes. The addition of health impact modelling results contributes to the discussion on what is involved in pedestrian and cyclist friendly futures. This approach suggests an alternative way of thinking about policies, both foregrounding the kind of social and economic changes which could make stronger, more effective policies possible and considering how policies might promote resilience in the context of such changes (e.g. large reductions in energy availability). The medical and public health communities have a responsibility to highlight the importance of health when considering alternative futures and should influence planners to move away from a car-dependent society.

Encouragingly we found a reduction in injuries, although under certain assumptions injuries increased and given the uncertainty about the mechanisms explaining non-linearity of risk [52] it is important that policies focus on reducing risk whilst increasing walking and cycling.

Sensitivity analysis highlights the importance of changes to who is walking and cycling. In the Netherlands, cycling is not only much more common than in England and Wales but it is relatively much more widespread at older ages. If cycling increased in England and Wales but did not become relatively more common amongst older people then the analysis indicates that the anticipated health impact would be smaller.

Decisions on investments in transport are often dominated by travel time savings [53]. Travel time savings are mainly achieved by increasing the speed of motorised transport. There is a good case for prioritising the health benefits from investments in transport over travel time savings benefits. Although considerable uncertainty remains around quantification of these health benefits they may still represent more tangible benefits than those from time savings, which will usually be taken first as improved accessibility for those using cars and then over time become changes in land use, with increased urban sprawl and the expectation of and requirement for longer travel distances woven into the urban fabric [53].

Current appraisal methods would typically try to compare health impacts, CO_2_ emissions, and time savings within a common monetised metric (e.g. [3]); however, it can be argued that such a measure does little to provide useful information for social decision making (e.g. [54]) Given the strong arguments that large emission reductions are necessary to reduce the risk of climate destabilisation, it might be more appropriate to evaluate how effective options are at achieving these reductions and the extent of any co-benefits or harms, rather than monetising reductions in CO2 emissions [55]. Beyond this, a case can be made for starting from normative goals of what healthy and low carbon transport systems should be like and then working backwards around the question of how to get there.

### Unanswered questions and future research

Different relative risk functions and modelling approaches produce very different results. Summing results from individual diseases produced considerably smaller results than those observed when directly estimating effects on all-cause mortality. Although physical activity may affect diseases not included in the model this is unlikely to explain the size of the difference. Future modelling and empirical research should investigate these differences and attempt to reduce uncertainty in this area.

The extent to which increases in walking and cycling would affect weight gain and obesity is uncertain but such mechanisms could represent an important health dimension that is not included in this model. Relatives risks were chosen, if possible, that adjusted for obesity, and thus health gains from reductions in obesity could be in addition to those reported in this paper.

Future modelling work should consider how we might achieve step-changes in active travel. There is an argument for going beyond focusing on marginal policy changes to investigation of the potential tipping points in transport systems that might lead to substantively different futures.

## Conclusion

Moving urban trips from car travel to walking and cycling can provide substantive benefits to population health and reduce transport related greenhouse gas emissions. The largest benefits are likely to be from changes to physical activity. The study suggests that total injuries need not go up with increased walking and cycling as long as there are sufficient reductions in motor vehicle distance and lower motor vehicle speeds. Policies to achieve a step-change in active travel and reduce use of motor vehicles should be supported.

## Supporting Information

Figure S1 **Schematic of physical activity model.** This figure illustrates the key data sources and stages in the physical activity module component of ITHIM implemented in Excel.
(TIF)

Figure S2 **Schematic of road traffic injury model.** This figure illustrates the key data sources and stages in the road traffic injury module component of ITHIM implemented in Excel.
(TIF)

Figure S3 **Schematic of air pollution model.** This figure illustrates the key data sources and stages in the air pollution module component of ITHIM implemented in Excel. (CRA: Comparative Risk Assessment)
(TIF)

Table S1 **Non-travel MET hours per week by quintile of active travel from the Health Survey for England 2008.** Estimated weekly MET values for activity from all the other domains for each quintile of walking and cycling activity in HSE within each demographic group. The quintile of active travel is based on estimated walking plus cycling time taken from the Health Survey for England 2008.
(DOCX)

Table S2 **Baseline Serious Injuries by victim mode and striking vehicle for minor roads in urban areas in England and Wales.** This table shows the estimated annual average number of serious injuries by the victim mode and striking vehicle in urban areas outside London between 2002 and 2008. All other data by severity (fatalities) and by road type (major roads and motorways) are available on request from the authors.
(DOCX)

Table S3 **PM 2.5 emission factors per km by vehicle type and road type.** Emission factors taken from the UK National Air pollution Emissions Inventory for 2008.
(DOCX)

Table S4 **CO_2_ emission factors, grams per vehicle kilometre.** Emission factors taken from the UK National Air pollution Emissions Inventory for 2008.
(DOCX)
