# Supplementary figures and images for: Health Impact Modelling of Active Travel Visions for England and Wales Using an Integrated Transport and Health Impact Modelling Tool (ITHIM)
Source: PLoS One. 2013 Jan 9;8(1):e51462. doi: 10.1371/journal.pone.0051462 (PMC3541403; doi:10.1371/journal.pone.0051462)

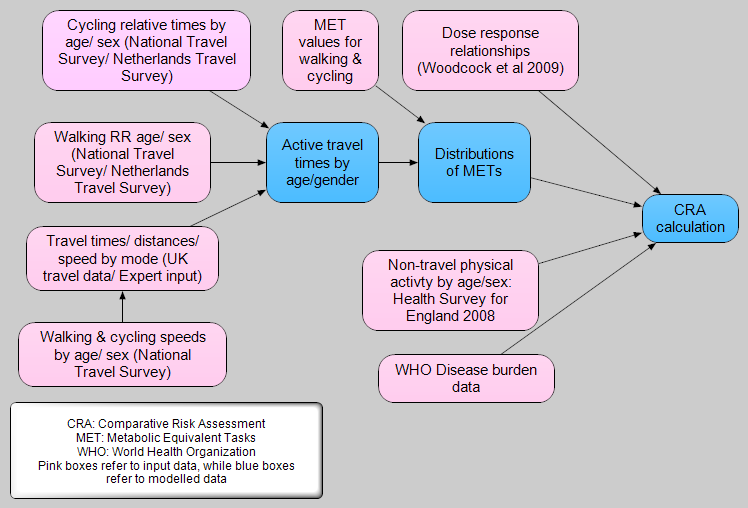

Supplement: Figure S1 — Schematic of physical activity model. This figure illustrates the key data sources and stages in the physical activity module component of ITHIM implemented in Excel. (TIF) [file pone.0051462.s001.tif]

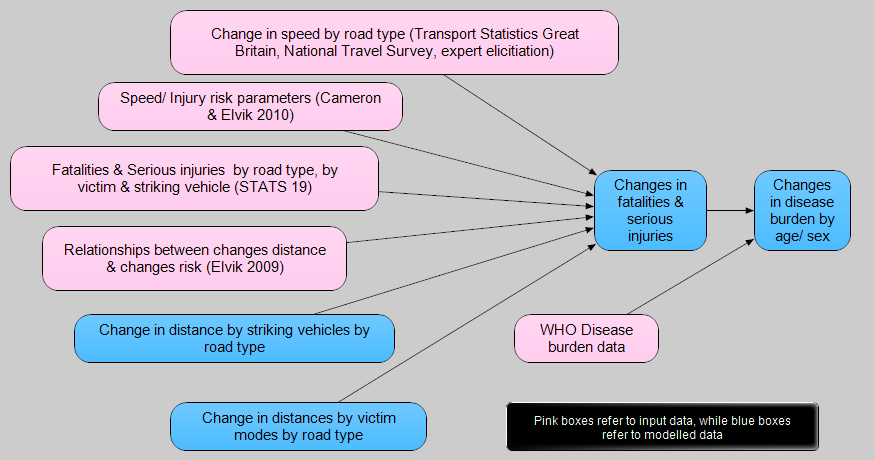

Supplement: Figure S2 — Schematic of road traffic injury model. This figure illustrates the key data sources and stages in the road traffic injury module component of ITHIM implemented in Excel. (TIF) [file pone.0051462.s002.tif]

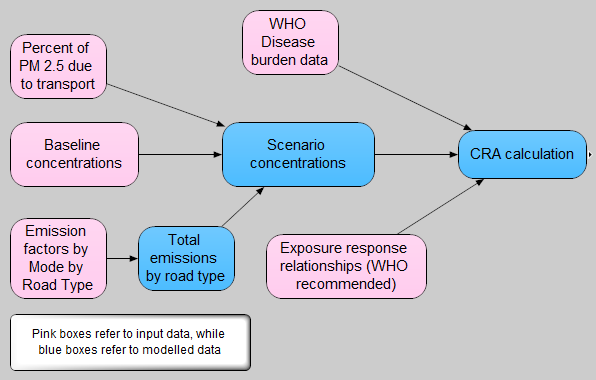

Supplement: Figure S3 — Schematic of air pollution model. This figure illustrates the key data sources and stages in the air pollution module component of ITHIM implemented in Excel. (CRA: Comparative Risk Assessment) (TIF) [file pone.0051462.s003.tif]
