# Supplementary material for: Health Impact Modelling of Active Travel Visions for England and Wales Using an Integrated Transport and Health Impact Modelling Tool (ITHIM)
Source: PLoS One. 2013 Jan 9;8(1):e51462. doi: 10.1371/journal.pone.0051462 (PMC3541403; doi:10.1371/journal.pone.0051462)
Supplement: Table S1 — Non-travel MET hours per week by quintile of active travel from the Health Survey for England 2008. Estimated weekly MET values for activity from all the other domains for each quintile of walking and cycling activity in HSE within each demographic group. The quintile of active travel is based on estimated walking plus cycling time taken from the Health Survey for England 2008. (DOCX) [file pone.0051462.s004.docx]

**TABLE S1: Non-travel MET hours per week by quintile of active travel from the Health Survey for England 2008**

|  | **Quintile of active travel** | | | | | |
| --- | --- | --- | --- | --- | --- | --- |
| Baseline | Age | 1 | 2 | 3 | 4 | 5 |
| men | 0-4 | 0 | 0 | 0 | 0 | 0 |
|  | 5-14 | 0 | 0 | 0 | 0 | 0 |
|  | 15-29 | 20 | 41 | 24 | 22 | 45 |
|  | 30-44 | 60 | 53 | 42 | 56 | 56 |
|  | 45-59 | 33 | 44 | 40 | 38 | 40 |
|  | 60-69 | 4 | 8 | 4 | 11 | 10 |
|  | 70-79 | 0 | 0 | 0 | 0 | 0 |
|  | 80+ | 0 | 0 | 0 | 0 | 0 |
| women | 0-4 | 0 | 0 | 0 | 0 | 0 |
|  | 5-14 | 0 | 0 | 0 | 0 | 0 |
|  | 15-29 | 5 | 11 | 16 | 8 | 17 |
|  | 30-44 | 16 | 23 | 17 | 17 | 18 |
|  | 45-59 | 20 | 20 | 20 | 23 | 27 |
|  | 60-69 | 0 | 0 | 0 | 0 | 3 |
|  | 70-79 | 0 | 0 | 0 | 0 | 0 |
|  | 80+ | 0 | 0 | 0 | 0 | 0 |
