# Supplementary material for: Health Impact Modelling of Active Travel Visions for England and Wales Using an Integrated Transport and Health Impact Modelling Tool (ITHIM)
Source: PLoS One. 2013 Jan 9;8(1):e51462. doi: 10.1371/journal.pone.0051462 (PMC3541403; doi:10.1371/journal.pone.0051462)
Supplement: Table S2 — Baseline Serious Injuries by victim mode and striking vehicle for minor roads in urban areas in England and Wales. This table shows the estimated annual average number of serious injuries by the victim mode and striking vehicle in urban areas outside London between 2002 and 2008. All other data by severity (fatalities) and by road type (major roads and motorways) are available on request from the authors. (DOCX) [file pone.0051462.s005.docx]

**TABLE S2: Baseline Serious Injuries by victim mode and striking vehicle for minor roads in urban areas in England and Wales (average per year 2002-2008)**

|  |  | Striking vehicle | | | | | | |
| --- | --- | --- | --- | --- | --- | --- | --- | --- |
|  |  | heavy goods vehicle | bus | light goods vehicle | car | motor bike | cycle | no other vehicle |
| Victim mode | walk | 53 | 147 | 145 | 2342 | 71 | 23 | 0 |
|  | cycle | 20 | 20 | 48 | 763 | 14 | 18 | 46 |
|  | motorbike | 20 | 19 | 60 | 885 | 43 | 0 | 268 |
|  | car | 63 | 53 | 120 | 1439 | 8 | 0 | 574 |
|  | light goods vehicle | 6 | 2 | 32 | 0 | 0 | 0 | 15 |
|  | bus | 2 | 45 | 0 | 0 | 0 | 0 | 69 |
|  | heavy goods vehicle | 8 | 0 | 0 | 0 | 0 | 0 | 6 |
