# Supplementary material for: Health Impact Modelling of Active Travel Visions for England and Wales Using an Integrated Transport and Health Impact Modelling Tool (ITHIM)
Source: PLoS One. 2013 Jan 9;8(1):e51462. doi: 10.1371/journal.pone.0051462 (PMC3541403; doi:10.1371/journal.pone.0051462)
Supplement: Table S3 — PM 2.5 emission factors per km by vehicle type and road type. Emission factors taken from the UK National Air pollution Emissions Inventory for 2008. (DOCX) [file pone.0051462.s006.docx]

**TABLE S3: PM 2.5 emission factors per km by vehicle type and road type**

|  |  | PM 2.5 g/km | | | |
| --- | --- | --- | --- | --- | --- |
|  |  | hot & cold start | break wear | tyre wear | total |
|  | Rigid HGVs | 0.077 | 0.011 | 0.012 | 0.100 |
|  | Artic HGVs | 0.120 | 0.007 | 0.025 | 0.152 |
|  | Buses | 0.104 | 0.017 | 0.014 | 0.134 |
|  | Motor cycle | 0.016 | 0.002 | 0.002 | 0.020 |
| All cars | urban | 0.016 | 0.003 | 0.005 | 0.024 |
|  | rural | 0.011 | 0.003 | 0.005 | 0.019 |
|  | Motorway | 0.016 | 0.003 | 0.005 | 0.024 |
| All LGVs | urban | 0.081 | 0.004 | 0.008 | 0.094 |
|  | rural | 0.045 | 0.004 | 0.008 | 0.058 |
|  | Motorway | 0.062 | 0.004 | 0.008 | 0.074 |
