# Supplementary material for: Health Impact Modelling of Active Travel Visions for England and Wales Using an Integrated Transport and Health Impact Modelling Tool (ITHIM)
Source: PLoS One. 2013 Jan 9;8(1):e51462. doi: 10.1371/journal.pone.0051462 (PMC3541403; doi:10.1371/journal.pone.0051462)
Supplement: Table S4 — CO2 emission factors, grams per vehicle kilometre. Emission factors taken from the UK National Air pollution Emissions Inventory for 2008. (DOCX) [file pone.0051462.s007.docx]

**TABLE S4: CO_2_ emission factors, grams per vehicle kilometre**

|  | CO_2_ emissions (g) per km |
| --- | --- |
| Bus | 958 |
| Cars urban | 222 |
| Cars motorway | 204 |
| Motorbike | 90 |
